# Concurrent Management of Prostate Cancer and Perianal Fistula: A Case Report, Treatment Considerations, and Review of Literature

**DOI:** 10.1155/criu/9964573

**Published:** 2026-08-21

**Authors:** Kwabena Boahen Asare, Lorraine Zaki, Bassem Zaki

**Affiliations:** ^1^ Department of Radiation Oncology and Applied Sciences, Dartmouth Hitchcock Medical Center, Lebanon, New Hampshire, USA, dartmouth-hitchcock.org; ^2^ Dartmouth College, Hanover, New Hampshire, USA, dartmouth.edu; ^3^ Touro College of Osteopathic Medicine, Middletown, New York, USA, touro.edu

**Keywords:** fistula-in-ano, image-guided radiation therapy, intensity-modulated radiation therapy, perianal fistula, prostate cancer, radiation therapy, staged surgery

## Abstract

**Background:**

Prostate cancer and pre‐existing perianal fistula are challenging conditions to manage concurrently as pelvic radiotherapy may raise concern for impaired wound healing, infection, or fistula progression. Although radiotherapy has traditionally raised concern as a risk factor for fistulization, this case suggests that selected patients with a pre‐existing perianal fistula may be treated with careful multidisciplinary planning and modern radiotherapy techniques.

**Case Presentation:**

A 70‐year‐old male diagnosed with favorable intermediate‐risk prostate cancer and a concurrent perianal fistula was treated with definitive radiation therapy and staged surgical treatment, respectively. The patient underwent intensity‐modulated radiation therapy (IMRT) with daily image‐guided radiation therapy (IGRT), receiving a total dose of 70 Gy in 28 fractions while minimizing radiation dose to the perianal region. Prior to radiation, a seton was placed to facilitate drainage and reduce infection risk from the fistula. Following radiotherapy, the patient underwent successful surgical fistulectomy without complications. At 26 months posttreatment, the patient remains free of biochemical recurrence and without recurrence of the perianal fistula.

**Conclusions:**

This case highlights the clinical importance of applying and coordinating established management principles to an uncommon and challenging scenario. Although seton placement, IMRT/IGRT, and fistula surgery are individually well‐established approaches, their sequencing in the setting of localized prostate cancer with a concurrent pre‐existing perianal fistula remains poorly described. This report offers a practical example of individualized, staged management in a complex clinical scenario for which standardized treatment pathways remain limited.

## 1. Background

Although prostate cancer is a common malignancy with well‐established treatment protocols, [1–5] the presence of concurrent anorectal conditions such as perianal fistula can complicate therapeutic decision‐making, particularly when radiation therapy is considered.

Perianal fistula, also known as fistula‐in‐ano is a nonmalignant anorectal condition characterized by pain in the anal and rectal areas, incontinence, and pus discharge [6]. It manifests as a chronic abnormal passage that connects the inner anal lining to nearby skin, forming a narrow channel with openings both inside and outside the anus [6]. This disorder is more common in men. About 12.3 per 100,000 men and 5.6 per 100,000 women are affected by this condition [7]. More than 90% of perianal fistulas originate from cryptoglandular sources [6], with the infection of the intersphincteric glands as the initiating event [8]. Even among experienced colorectal surgeons, surgically treating perianal fistulas can be challenging, resulting in the ongoing debate over the optimal treatment approach [6].

The presence of a pre‐existing perianal fistula may complicate consideration of pelvic radiotherapy due to concerns regarding tissue injury, infection, impaired healing, and fistula progression [9]. To our knowledge, there have been no reported cases of administering radiation therapy for prostate cancer in patients with a pre‐existing, unrelated perianal fistula. However, here, we present a case involving a prostate cancer patient who underwent both radiation therapy and surgical treatment for a pre‐existing, unrelated perianal fistula. Our report adds to the growing body of literature on optimal surgical treatment methods for perianal fistula while providing valuable insights into the management of prostate cancer with concurrent perianal fistula.

## 2. Literature Review

A comprehensive literature review was conducted using PubMed to identify publications relevant to prostate cancer management in the setting of perianal fistula. Search terms included combinations of keywords such as “prostate cancer,” “localized prostate cancer,” “perianal fistula,” “fistula‐in‐ano,” “radiation therapy,” “intensity‐modulated radiation therapy,” “image‐guided radiation therapy,” “radical prostatectomy,” “brachytherapy,” “seton,” and “fistulectomy.” The search was limited to papers written in English. Although we found several results for prostate cancer and perianal fistula individually, we found no publications that listed a concurrence of prostate cancer and perianal fistula in patients. We then reviewed relevant publications and their references.

## 3. Case Presentation

A 70‐year‐old Caucasian male was diagnosed with favorable intermediate‐risk prostate cancer, characterized by adenocarcinoma, Grade II, Gleason score of 7 (3 + 4) and a PSA of 9.9. A multiparametric MRI in May 2022 revealed a suspicious lesion in the prostate (PI‐RADS 5), leading to a transrectal ultrasound and biopsy in June 2022, which confirmed prostate adenocarcinoma involving 50% of a single core. His cancer was staged as T1c N0 M0, indicating no nodal or metastatic involvement. Overall, these findings were consistent with favorable intermediate‐risk localized prostate adenocarcinoma. Androgen deprivation therapy was not administered, given the favorable intermediate‐risk features and the plan for definitive moderately hypofractionated external‐beam radiotherapy alone.

The principal clinical challenge was determining how to deliver definitive prostate cancer treatment while minimizing the risk of fistula progression, recurrent infection, impaired wound healing, or disruption of subsequent fistula repair.

In addition to prostate cancer, the patient has a significant medical history of rheumatoid arthritis (RA) and hypertension, both of which are managed with a comprehensive medication regimen, including prednisone, hydroxychloroquine, and abatacept. However, abatacept was discontinued for the patient until after his radiation therapy.

The patient also has a history of alcohol use and tobacco abuse. These lifestyle factors have likely contributed to his chronic health issues, including degenerative joint disease, alcoholic liver disease, and chronic pain in his right ankle.

**Figure 1:**
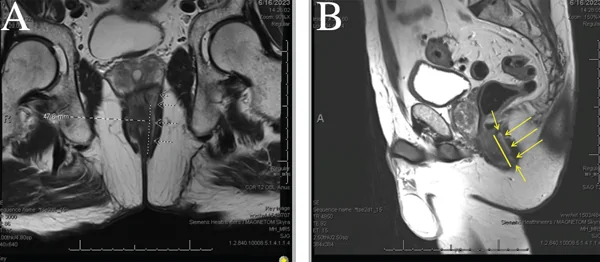
MRI scans showing positions of prostate and perianal fistula. (A) Coronal MRI image demonstrating perianal fistula (marked by arrows) of 47.6 mm in length. (B) Sagittal MRI image showing perianal fistula, marked by yellow arrows.

The patient also had a history of multiple perineal abscesses, likely involving the left ischiorectal fossa, which may have contributed to the development of an intersphincteric fistula. Clinical examination demonstrated an external fistula opening in the left posterior anal canal with a posterior midline defect at the dentate line, without masses or other abnormal findings. Pelvic MRI demonstrated a left‐sided intersphincteric fistula extending cranially from the 3:00 position to caudally at the 5:00 position and continuing to the skin (Figure 1). The fistula measured approximately 5 cm in length. At the apex of the fistula, there was a 1.8 × 1.7 × 2.0 cm heterogeneous collection containing fluid and air with peripheral enhancement, consistent with an abscess at the level of the anorectal junction. The fistula was comma‐shaped in the axial plane, traveled within the intersphincteric space, and measured 1.4 cm in maximal transverse dimension. He considered his treatment options and ultimately refused surgery in favor of radiation therapy.

Before drainage and seton placement, the patient had a history of multiple perianal/perineal abscesses, indicating active or recurrent fistula‐associated infection risk prior to definitive prostate cancer treatment.

As the first step in the staged management plan, a silastic vessel loop seton was passed through the fistula tract and secured to itself with 0‐silk ligatures before radiotherapy and was maintained throughout the radiation course. The purpose of seton placement was to preserve drainage of the fistula tract, reduce the risk of recurrent abscess or uncontrolled local infection during prostate radiotherapy, and provide a clinically visible marker of the fistula region during treatment planning.

**Figure 2:**
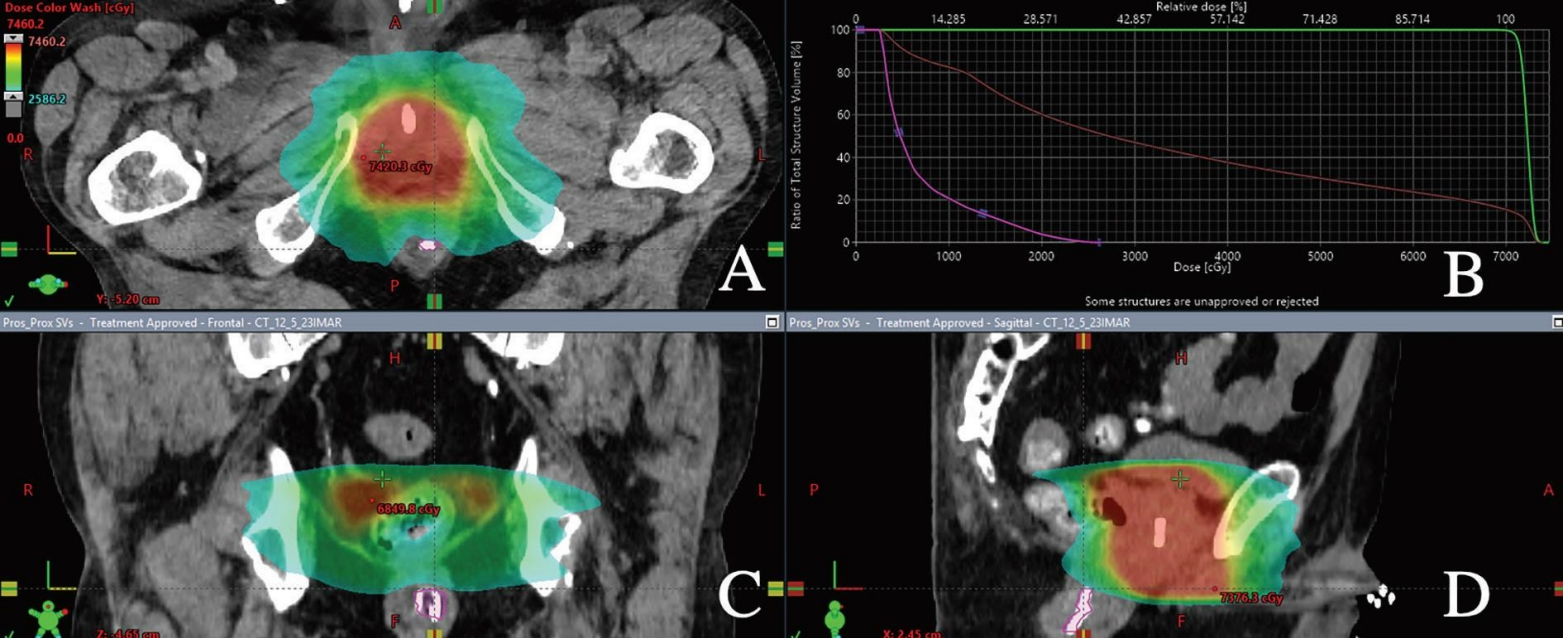
Multipanel figure indicating various aspects of radiotherapy planning. Green organ represents prostate. Small pink region represents the position of the perianal fistula marked by seton placement. (A) Coronal CT scan with color wash indicating positions of the prostate and perianal fistula. (B) Dose‐volume histogram (DVH) illustrating the relative dose percentages received by the prostate (green curve) and perianal region (pink curve) during radiotherapy. (C) Coronal CT scan showing the perianal region. (D) Sagittal CT scan showing positions of the prostate and perianal fistula.

Given the proximity of the fistula tract to the prostate treatment region, radiotherapy planning was designed to maintain definitive target coverage while limiting incidental dose to the perianal tissues whenever feasible. To support reproducibility of the treatment approach, key planning and dose parameters are summarized below:

The perianal fistula and seton were contoured as a dedicated avoidance structure based on direct visualization on fused MRI and CT images obtained at the time of simulation (Figure 2). All organ‐at‐risk (OAR) dose constraints were met, including rectal doses of V5900 cGy 24.397%, V6400 cGy 21.037%, and V6900 cGy 16.682%, and bladder doses of V6400 cGy 25.135%, V6900 cGy 21.113%, and V7400 cGy 0.013%. Femoral head dose constraints were satisfied, with D0.035 cc of 2665 cGy to the left femur and 2879.1 cGy to the right femur. The bowel space received a maximum dose (D0.035 cc) of 1585.8 cGy, and the penile bulb received a mean dose of 4595.5 cGy. Dose–volume histogram analysis of the contoured fistula/seton demonstrated a minimum dose of 244.4 cGy, a maximum dose of 2644.3 cGy, and a mean dose of 697.9 cGy. Pretreatment IMRT quality assurance was performed prior to delivery, with gamma analysis demonstrating 99.9% of sampled points within 3.0%/2.0 mm of the predicted dose distribution. Daily image‐guided radiation therapy (IGRT) was conducted following immobilization using kV imaging with fiducial marker matching; three gold fiducial markers had been placed in the prostate under ultrasound guidance prior to simulation.

The patient was treated with definitive radiation therapy, receiving 70 Gray in 28 fractions using intensity‐modulated radiation therapy (IMRT) with daily IGRT. He tolerated radiation well, with a good biochemical response, and his PSA levels began to decline within 3 months.

Three months after completing radiation treatment for prostate cancer, he underwent surgical treatment for the perianal fistula. At the time of repair, exam under local anesthesia using a perianal block showed the seton was intact, with no signs of an active abscess or fistula in the left posterior quadrant. Digital examination of the anal canal showed normal tone, no masses, and no blood. Anoscopic inspection revealed normal mucosa.

The surgical treatment for this perianal fistula involved the following steps. The seton was divided with scissors and removed. A probe was passed from the external opening of the fistula to the internal opening at the anal verge. There was minimal muscle involvement, as the fistula appeared to be predominantly superficial. It was carefully divided with cautery, and hemostasis was ensured. The tract was gently debrided, and the perineum was then dressed with fluff gauze, ABD pads, and mesh briefs.

This fistulectomy was successful, and the patient tolerated it well without complications. Following fistulectomy, the patient was managed with sitz baths twice daily, short‐course oral analgesics, and local hygiene measures. No antibiotics were required, and the wound healed by secondary intention over several weeks. At the last follow‐up, 26 months after completion of radiotherapy, the patient was clinically well, with no wound‐healing complications, no recurrent infection, no fecal incontinence, and no fistula recurrence. His most recent PSA, measured in April 2026, was 0.45 ng/mL. He remained free of biochemical recurrence by the Phoenix definition, defined as PSA nadir +2 ng/mL.

## 4. Discussion

It has been estimated that 10% of perianal abscesses are associated with sexually transmitted infections, tuberculosis, radiotherapy, Crohn′s disease (CD), inflammation, and other conditions [10]. However, 90% of perianal abscesses are caused by cryptoglandular infections [6, 10]. The most widespread etiology of fistula‐in‐ano is that the perianal infection progresses downward to the wall of the anal canal, penetrating any opening or wound in the anal area [8, 11]. Once the infected tract forms, it is sustained by fecal contamination [8, 11].

### 4.1. Genetic Considerations

Although genetic predisposition is not directly relevant to this patient′s presentation, we briefly reviewed known genetic associations as some reports suggest overlap between inflammatory pathways involved in fistula formation and pathways implicated in prostate carcinogenesis. We found no other reports in the literature that comment on the management or genetic predisposition of concurrent treatment of perianal fistula and prostate cancer without the presence of inflammatory bowel disease [12–16]. Existing literature describes genetic risk factors for prostate cancer and genetic associations with perianal fistula primarily in the setting of Crohn′s disease. NOD2 variants have been associated with Crohn′s disease and perianal fistulizing disease, whereas BRCA1/2, HOXB13, and other inherited variants have been associated with prostate cancer susceptibility [15–20]. However, these associations appear to represent distinct disease pathways rather than an established biologic link between cryptoglandular perianal fistula and localized prostate cancer.

### 4.2. Radiation and Perianal Fistula

In patients with perianal fistula, pelvic radiotherapy may raise clinical concern as tissue injury, inflammation, fibrosis, and impaired healing could worsen the fistula‐related morbidity or complicate later surgical repair [21]. These concerns are particularly relevant when the fistula tract lies near the anticipated treatment field. For this reason, careful treatment selection, drainage of active fistula disease, radiotherapy planning, and coordination with colorectal surgery are central to management.

For the patient described in this report, the field of radiation had minimal overlap with the field of the perianal fistula, allowing the perianal area to be designated as an avoidance structure during treatment planning. Dose–volume histogram analysis of the contoured fistula/seton region demonstrated a minimum dose of 244.4 cGy, maximum dose of 2644.3 cGy, and mean dose of 697.9 cGy; therefore, the maximum dose to this region was approximately 26.4 Gy (Figure 2B). IMRT was employed to enhance dose conformality and minimize dose to the fistula while maintaining adequate coverage of the prostate target. IGRT using gold coils fiducials further supported target localization and limited unnecessary expansion of treatment margins.

### 4.3. RA and Radiation Therapy

This patient′s course was complicated by their pre‐existing RA. RA can complicate the administration of radiation therapy due to several reasons. Studies have shown that lymphocytes from RA patients demonstrate increased radiosensitivity and slower DNA damage repair responses compared to those from healthy individuals [22]. Whereas the vast majority of RA patients do not show increased radiation toxicity, a few cases have been reported where RA patients demonstrated significant radiation‐induced toxicity [23].

Moreover, the autoimmune nature of RA meant that the patient′s immune system was compromised, increasing their risk of infections and delayed healing. The chronic inflammatory nature of RA, coupled with the immune system dysregulation it triggers, also increases the risk of scarring and fibrosis following radiation exposure [21].

The pathophysiology of this process involves persistent activation of inflammatory pathways, which leads to the release of profibrotic cytokines such as transforming growth factor‐beta (TGF‐*β*) [21]. This cytokine promotes the proliferation and activation of fibroblasts into myofibroblasts, which then secrete excessive extracellular matrix components, leading to fibrosis [21]. Additionally, the chronic inflammation associated with RA leads to the accumulation of immune cells, such as macrophages and T cells, which further contribute to tissue damage and fibrosis [21]. Oxidative stress and cellular senescence induced by ionizing radiation exacerbate these effects, making tissues more prone to scarring and fibrotic changes when exposed to radiation [21].

Given these complexities, special adaptations were necessary to ensure the patient′s safety and efficacy of the treatment. To reduce immunosuppression and enhance the patient′s treatment outcome, one of their immunosuppressive medications (abatacept) was discontinued until after their radiation treatment.

Abatacept is an immunosuppressive medication commonly used to treat autoimmune conditions like RA. As T cells are crucial for the maintenance and onset of RA, abatacept functions by inhibiting T‐cell activation, thus managing RA [24]. Radiation therapy on the other hand is used to target and destroy cancer cells. The immune‐suppressing functionality of abatacept risks interfering with the body′s ability to mount an effective immune response against the cancer cells targeted by radiation. To reduce interference, abatacept was discontinued to enhance the effectiveness of radiation therapy for our patient′s prostate cancer.

## 5. Conclusions

The concurrent management of localized prostate cancer (with radiation therapy) and perianal fistula presents a complex clinical challenge, particularly given concerns regarding fistula progression, impaired wound healing, and infectious complications. In this case report, favorable intermediate‐risk prostate cancer was treated with definitive radiation therapy, followed by surgical management for a coexisting perianal fistula. Careful treatment planning, including efforts to minimize radiation exposure to the perianal region, allowed definitive prostate radiotherapy to be delivered while maintaining fistula control.

Our report highlights the importance of multidisciplinary coordination and individualized treatment sequencing in patients with concurrent prostate cancer and perianal fistula. Although broad conclusions regarding safety or wider application or generalizability cannot be drawn from a single case, this experience offers a practical example of how modern radiotherapy planning and staged surgical management may be integrated in a complex clinical setting. Further case reports with longer follow‐up are needed to better define patient selection criteria, technical considerations, and long‐term outcomes.

## 6. Limitations

First, given that the report concerns a single patient, it provides only preliminary insight and a foundation for future evaluation rather than firm evidence of safety or efficacy. Second, although the 26‐month follow‐up period provides useful early clinical information, longer surveillance is still needed to evaluate late radiation toxicity, delayed wound‐healing complications, fistula recurrence, and biochemical control. Third, the patient′s RA and history of immunosuppressive therapy introduce clinical factors that may influence radiation tolerance and wound healing. Hence, results may differ in individuals with a simpler medical profile. Broader studies across multiple institutions with longer surveillance and patient populations free of major confounding conditions would offer a more authoritative basis for assessing safety, refining dose parameters, and developing consistent treatment strategies.

**Nomenclature**
ABD: abdominal (as in ABD pads)
BRCA1/2 mutations: breast Cancer 1 and 2 gene mutations
CD: Crohn′s disease
DVH: dose‐volume histogram
HOXB13: homeobox B13 gene
IGRT: image‐guided radiation therapy
IMRT: intensity‐modulated radiation therapy
MRI: magnetic resonance imaging
NOD2: nucleotide‐binding oligomerization domain‐containing protein 2
PI‐RADS: prostate imaging reporting and data system
PSA: prostate‐specific antigen
RA: rheumatoid arthritis
SNP: single nucleotide polymorphism
TGF‐*β*
: transforming growth factor‐beta

## Author Contributions

K.B.A. contributed to literature review, data analysis, figure preparation, and drafting of the manuscript. L.Z. contributed to the literature review pertaining to genetic considerations of the case. B.Z. supervised clinical case management, provided critical revisions, and shaped the report.

## Funding

No funding was received for this manuscript.

## Disclosure

All authors read and approved the final manuscript.

## Ethics Statement

The patient provided informed consent for all clinical procedures, including seton placement, radiation therapy, and subsequent surgical management, as part of routine clinical care. For publication, the patient provided separate written informed consent after being counseled on the purpose of the report, the information to be disclosed, and the measures taken to protect anonymity. No identifiable images or details are included. Consistent with Dartmouth Health policy, the IRB determined that single‐patient case reports with fully deidentified information do not require formal IRB review.

## Consent

The patient gave his full consent for publication, and written informed consent was obtained from the patient for anonymized information to be published in this article.

## Conflicts of Interest

The authors declare no conflicts of interest.

## Data Availability

All data supporting the conclusions of this case report are included within the article. The imaging figures used in the report are available from the corresponding author upon reasonable request, with appropriate safeguards to protect patient privacy and confidentiality. No additional datasets were generated or analyzed during the current study.
